# Mannosylation in *Candida albicans*: role in cell wall function and immune recognition

**DOI:** 10.1111/mmi.12426

**Published:** 2013-11-08

**Authors:** Rebecca A. Hall, Neil A. R. Gow

**Affiliations:** ^1^ Aberdeen Fungal GroupSchool of Medical SciencesUniversity of AberdeenAberdeenAB252ZDUK

## Abstract

The fungal cell wall is a dynamic organelle required for cell shape, protection against the environment and, in pathogenic species, recognition by the innate immune system. The outer layer of the cell wall is comprised of glycosylated mannoproteins with the majority of these post‐translational modifications being the addition of *O*‐ and *N*‐linked mannosides. These polysaccharides are exposed on the outer surface of the fungal cell wall and are, therefore, the first point of contact between the fungus and the host immune system. This review focuses on *O*‐ and *N*‐linked mannan biosynthesis in the fungal pathogen *Candida albicans* and highlights new insights gained from the characterization of mannosylation mutants into the role of these cell wall components in host–fungus interactions. In addition, we discuss the use of fungal mannan as a diagnostic marker of fungal disease.

## Introduction

*Candida albicans* is an opportunistic fungal pathogen of humans, which is part of the natural flora of the oral, genital and gastrointestinal tracts. The maintenance of colonization over dissemination is achieved through an intricate balance of fungal proliferation and host immune recognition and control. During periods of immune suppression, caused by chemotherapy, trauma, age and cancer, *C. albicans* is able to overcome the immune system, disseminate and cause life‐threatening systemic disease. The associated mortality rates of systemic fungal disease are reported to be up to 40%, which is higher than that reported for most bacterial infections (Almirante *et al*., 2005; Klevay *et al*., 2008; Leroy *et al*., 2009). It is also a frequent mucosal pathogen, with more than 75 million women suffering from vaginitis each year (Sobel, 2007).

The interplay between *C. albicans* and the host immune system is largely mediated by components of the fungal cell wall including mannans, β‐glucans and chitin. The structural organization of the fungal cell wall has been extensively reviewed elsewhere (Bowman and Free, 2006; Latgé, 2007; Gow and Hube, 2012), but comprehensive reviews on fungal mannan biosynthesis are limited. This review focuses on *O*‐ and *N*‐mannan biosynthesis, the role(s) of mannans in innate immune recognition, and the use of fungal mannan as a diagnostic marker for invasive candidaemia.

## The cell wall

The fungal cell wall is a dynamic structure important for maintaining cell shape, protection and stability against environmental stresses and outwardly directed turgor pressure and for immunogenicity. The cell wall must be physically robust, but also flexible enough to permit cell expansion, cell division and morphogenesis. The wall must also be permeable to allow egress of secreted proteins and the import of solutes, but sufficiently impermeable to protect the inner skeletal layer from environmental hydrolases. The cell wall is comprised of three major polysaccharides, chitin, glucans and mannans. In *C. albicans,* these polysaccharides are organized as two layers: an inner skeletal layer of chitin and β1,3‐linked glucan and an outer layer of β1,6‐glucan and cell wall proteins anchored to the skeletal layer via a glycosylphosphatidylinositol (GPI) remnant. These proteins include cell wall remodelling enzymes involved in cell wall biogenesis (Douglas *et al*., 1997; Dünkler *et al*., 2005), modification of the properties of the nascent and mature polysaccharides, and proteins essential for adhesion (Buurman *et al*., 1998; Hoyer, 2001) and biofilm formation (Nobile *et al*., 2006; Zhao *et al*., 2006), all of which influence the pathogenesis of the organism. The cell wall and secreted proteins of *C. albicans* are highly decorated with elaborate carbohydrate structures comprised of α‐ and β‐linked mannose units referred to as mannoproteins. Mannose sugars are incorporated into three structures: linear *O*‐linked mannan, highly branched *N*‐linked mannan and phospholipomannan. Protein mannosylation occurs during protein synthesis in the endoplasmic reticulum (ER) and is further elaborated as the protein is passed through the Golgi apparatus. Initially, sugars (i.e. mannose and glucose) are added to dolichol phosphate acceptors, from which are then incorporated into *C*‐, *N*‐, *O*‐mannosylation, as well as GPI anchors. On the other hand, in the Golgi, the donor of mannosyl residues is GDP‐mannose. Initiation of mannosylation in *C. albicans* has been reviewed elsewhere (Mora‐Montes *et al*., 2009), and this review will focus on the transglycosylases involved in the elaboration of *O*‐ and *N*‐mannan structures.

## *C. albicans* mannosylation mutants

**Figure 1:**
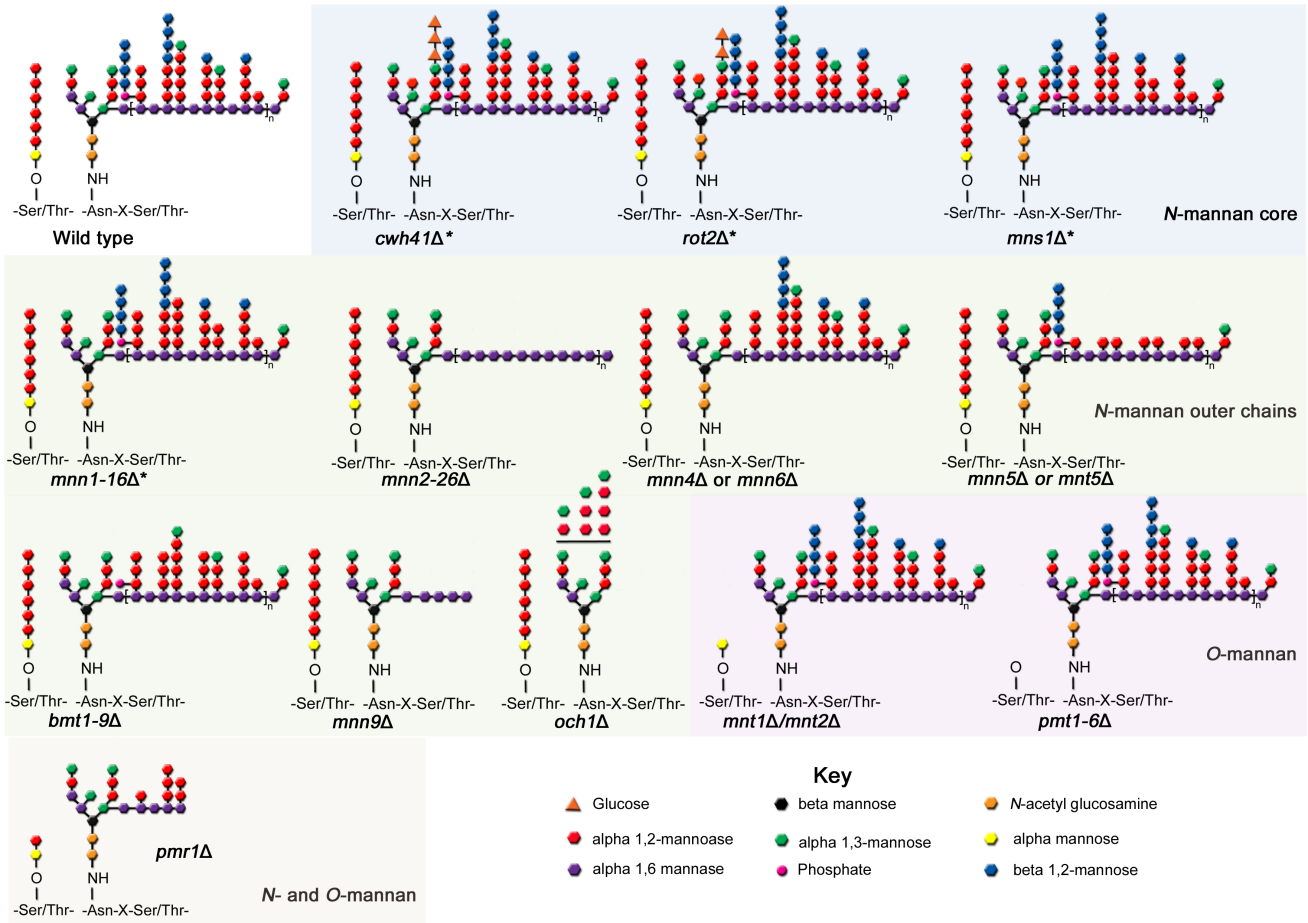
*N*‐ and *O*‐linked glycosylation structures of the *C. albicans* mannosylation mutants. Asterisks highlight structures, which are predicted from comparisons with *S. cerevisiae*, but have not yet been experimentally determined for *C. albicans.*

Studies exploring the role(s) of mannosylation in fungal biology and virulence have been informed by the creation of a series of *C. albicans* mannosylation mutants with truncations in the normal wild‐type structures of both *O*‐ and *N*‐linked mannan. Because these mutants express stably altered mannan structures on their cell surface (Fig. 1), these mutants have been used as tools to explore the importance of specific mannan epitopes on cell function, pathogenesis and immune recognition (Table 1).

**Table 1:** Summary of the phenotypes for the *C. albicans* mannosylation mutants

	Gene	Activity	Mutant phenotype							References
			Growth	Cell Wall	Sensitivity	Morphology	Adhesion	Phagocytosis	Virulence	
*O*‐mannan	*PMT1*	Required for initiation of *O*‐glycosylation	Biofilm formation reduced. Required for growth with *PMT4*	Reduced mannan, increased β1,3‐glucan	Increased sensitivity to Congo red, CFW, SDS, heat stress and antifungals	Hyphal growth reduced	Epithelial adhesion reduced		Reduced in HDC, RHE and oral mucosal models	Timpel *et al*. (1998); Prill *et al*. (2005); Rouabhia *et al*. (2005); Peltroche‐Llacsahuanga *et al*. (2006); Murciano *et al*. (2011)
	*PMT2*	Required for initiation of *O*‐glycosylation	Essential for viability, Biofilm formation reduced^a^		Increased sensitivity to Congo red, CFW, caffeine, heat stress and antifungals^a^	Hyphal growth reduced^a^		Normal^a^	Reduced in murine systemic infection model^a^	Prill *et al*. (2005); Rouabhia *et al*. (2005); Peltroche‐Llacsahuanga *et al*. (2006); Corbucci *et al*. (2007)
	*PMT4*	Required for initiation of *O*‐glycosylation	Biofilm formation reduced. Required for growth with *PMT1*	Reduced mannan, increased β1,3‐glucan	Increased sensitivity to antifungals	Hyphal growth reduced			Reduced in RHE and murine systemic infection model	Prill *et al*. (2005); Rouabhia *et al*. (2005)
	*PMT5*	Required for initiation of *O*‐glycosylation	Normal	Normal	Normal	Normal		Uptake normal, but cells are not killed by neutrophils	Reduced damage in oral mucosal models	Prill *et al*. (2005); Rouabhia *et al*. (2005); Corbucci *et al*. (2007)
	*PMT6*	Required for initiation of *O*‐glycosylation	Normal	Normal	Normal	Hyphal growth reduced	Epithelial adhesion reduced		Reduced damage in RHE model	Timpel *et al*. (2000); Prill *et al*. (2005); Rouabhia *et al*. (2005)
	*MNT1* *MNT2*	Addition of α1,2‐mannose	Cell separation defect in the double mutant	Reduced *O*‐mannan in double mutant	Increased sensitivity to CFW (*mnt1*Δ and double mutant only), double mutant has increased sensitivity to SDS	Reduced hyphal formation in the double mutant	Reduced	Neutrophil Uptake reduced, macrophage uptake increased	Reduced in murine systemic infection model (double mutant only)	Munro *et al*. (2005); McKenzie *et al*. (2010); Sheth *et al*. (2011)
*N*‐mannan	*CWH41*	Removes terminal α1,2 linked glucose from core	Reduced growth rate, increased flocculation	Reduced PM, mannan and glucan, increased chitin and protein	Increased sensitivity to Congo red, CFW, SDS and antifungals	Hyphal growth reduced			Reduced in murine infection model	Mora‐Montes *et al*. (2007)
	*ROT2*	Removes the two α1,3 linked glucose units from core	Reduced growth rate, increased flocculation	Reduced PM, mannan and glucan, increased chitin and protein	Increased sensitivity to Congo red, CFW, SDS and antifungals	Hyphal growth reduced			Reduced in murine infection model	Mora‐Montes *et al*. (2007)
	*MNS1*	Removes α1,2 mannose from core	Reduced growth rate, increased flocculation	Reduced PM	Increased sensitivity to Congo red and CFW	Normal		Macrophage uptake increased	Reduced in murine infection model	Mora‐Montes *et al*. (2007); McKenzie *et al*. (2010)
	*OCH1*	Addition of initial α1,6‐mannose	Increased cell size, decreased growth rate, cell separation defect	Reduced PM and mannan, Increased chitin and glucan	Increased sensitivity to Congo red, CFW, SDS, heat stress and antifungals	Hyphal growth reduced	Epithelial adhesion reduced	Neutrophil uptake reduced	Reduced in murine infection model	Bates *et al*. (2006); Murciano *et al*. (2011); Sheth *et al*. (2011)
	*MNN1* *MNN12* *MNN13* *MNN14* *MNN15* *MNN16*	Addition of terminal α1,3‐mannan	Normal	Extended PM chains *mnn14*Δ only)	Increased sensitivity to SDS and antifungals (*mnn14*Δ only)	Reduced hyphal growth in response to pH, temperature and Lee's and spider media (*mnn14*Δ only)			Reduced in murine infection model (*mnn14*Δ only)	Bates *et al*. (2013)
	*MNN2* *MNN21* *MNN22* *MNN23* *MNN24* *MNN26*	Addition of initial α1,2‐mannan to α1,6 backbone	Reduced growth rate and increased flocculation in double, triple, quintuple and sextuple mutants	*N*‐mannan and PM severely truncated in double, triple, quintuple and sextuple mutants. Chitin content increased	Increased sensitivity to Congo red, CFW and SDS,	Delayed hyphal growth (*mnn22*Δ, quintuple and sextuple mutants)			Reduced in *Galleria mellonella* and murine infection models	Bai *et al*. (2006); Hall *et al*. (2013)
	*MNN4*	Positive regulator of *MNN6*	Normal	Reduced PM	Normal	Normal		Neutrophil and macrophage uptake reduced	Normal	Hobson *et al*. (2004); McKenzie *et al*. (2010); Sheth *et al*. (2011)
	*MNN9*	Elaboration of the α1,6‐mannose backbone	Reduced growth rate, increased flocculation	Reduced mannan content	Increased sensitivity to antifungals	Hyphal growth reduced	Epithelial adhesion reduced			Southard *et al*. (1999); Murciano *et al*. (2011)
	*BMT1‐9*	Addition of β1,2‐ mannose	Normal	Normal	Normal	Normal			Normal	Mille *et al*. (2008)
	*MNT3‐5*	Addition of α1,2 mannose to outer chain	Normal, increased flocculation in multiple mutant	Reduced PM	Multiple mutant shows increased sensitivity to CFW, SDS and antifungals	Normal		Reduced macrophage uptake	Multiple mutant shows reduced virulence in murine infection model	McKenzie *et al*. (2010); Mora‐Montes *et al*. (2010)
PLM	*MIT1*	Addition of α‐mannan to lipid	Normal	No PLM, less β‐mannose in PM	Increased sensitivity to calcium and SDS	Normal		Increased susceptibility to phagocytosis	Reduced in murine infection model	Mille *et al*. (2004)
Other enzymes	*PMR1*	Transport of Ca^2+^/Mn^2+^ into Golgi	Normal	Reduced PM and *O‐*mannan	Increased sensitivity to Congo red, CFW, heat stress and antifungals	Delayed	Normal	Reduced neutrophil and macrophage uptake	Reduced in murine infection model	Bates *et al*. (2005); McKenzie *et al*. (2010); Sheth *et al*. (2011)

**a.** Phenotype of the heterozygous mutant.

### *O*‐mannosylation mutants

As discussed above, the *C. albicans O*‐mannan is a simple linear carbohydrate comprised of a series of α1,2‐linked mannose units (typically, 1–5 residues). The initial α‐mannose residue is attached to the hydroxyl group of serine/threonine residues through the actions of *PMT1, PMT2, PMT4, PMT5* and *PMT6* (Prill *et al*., 2005). Mnt1 and Mnt2 are partially redundant α1,2‐mannosyltransferases required for the addition of the first and second α1,2‐mannose units onto the α‐mannose (Munro *et al*., 2005). Deletion of *MNT1* and *MNT2* alone, or in combination, results in truncation of the *O*‐mannan (Buurman *et al*., 1998; Munro *et al*., 2005). Recent biochemical characterization of the MNT gene family suggests that *MNT1* may be required for further elaboration of the *O*‐mannan chain (Díaz‐Jiménez *et al*., 2012). Deletion of the PMT gene family, and *MNT1* and *MNT2* reduced the capacity for biofilm formation and resulted in increased sensitivity to cell wall perturbing agents such as Calcofluor White, Congo Red and SDS (Table 1), suggesting that *O*‐mannosylation is important for the general integrity of the cell wall (Timpel *et al*., 1998; Munro *et al*., 2005; Prill *et al*., 2005; Peltroche‐Llacsahuanga *et al*., 2006). Although a significant amount of redundancy is expected between the PMT family members, *PMT2* is the only member that has been shown to be essential for viability (Prill *et al*., 2005), suggesting that Pmt2 may play additional roles compared to the other family members. Likewise, Pmt1 and Pmt6 are required for the adhesive properties of the fungus to epithelial cells (Timpel *et al*., 1998; 2000; Murciano *et al*., 2011). All mutants involved in the biosynthesis of *O*‐mannan that have been studied show attenuated virulence in the murine systemic infection model, and most also have adhesion defects (Buurman *et al*., 1998; Timpel *et al*., 1998; Munro *et al*., 2005; Rouabhia *et al*., 2005) confirming the importance of *O*‐mannan in fungus‐host interactions (Table 1).

### *N*‐mannosylation mutants

#### *N*‐mannan core

The core structure of *N*‐mannan is a dolichol pyrophosphate anchored oligosaccharide comprised of three glucose, nine mannose and two *N*‐acetylglucosamine residues (Glc_3_Man_9_GlcNAc_2_). After attachment to asparagine residues within the polypeptide chain via the OST complex (Kelleher and Gillmore, 2006), this oligosaccharide is processed in the endoplasmic reticulum by three glycosidases (Cwh41, Rot2 and Mns1). These glycosidases remove the three terminal glucose units and one additional α1,2‐mannose units, forming the mature core (Man_8_GlcNAc_2_). The processed core is similar in structure in all eukaryotes, but the pattern of elaboration of the outer *N*‐mannan chains is fungal specific. Prevention of core processing by deletion of these genes not only affects the structure of the core, but also alters the structure of the outer chain branched *N*‐mannan (Mora‐Montes *et al*., 2007), suggesting that these processing steps are key regulators of *N*‐mannan biosynthesis. Deletion of *MNS1*, *CWH41* and *ROT2* results in increased flocculation, decreased growth and lower phosphomannan content (Mora‐Montes *et al*., 2007). These changes in cell wall composition also result in reduced secretion of pro‐inflammatory cytokines from human monocytes, correlating with attenuated virulence in the murine model of systemic candidiasis (Mora‐Montes *et al*., 2007). Therefore, full processing of the core *N*‐mannan is important for virulence (Table 1).

#### Branched *N*‐mannan

**Figure 2:**
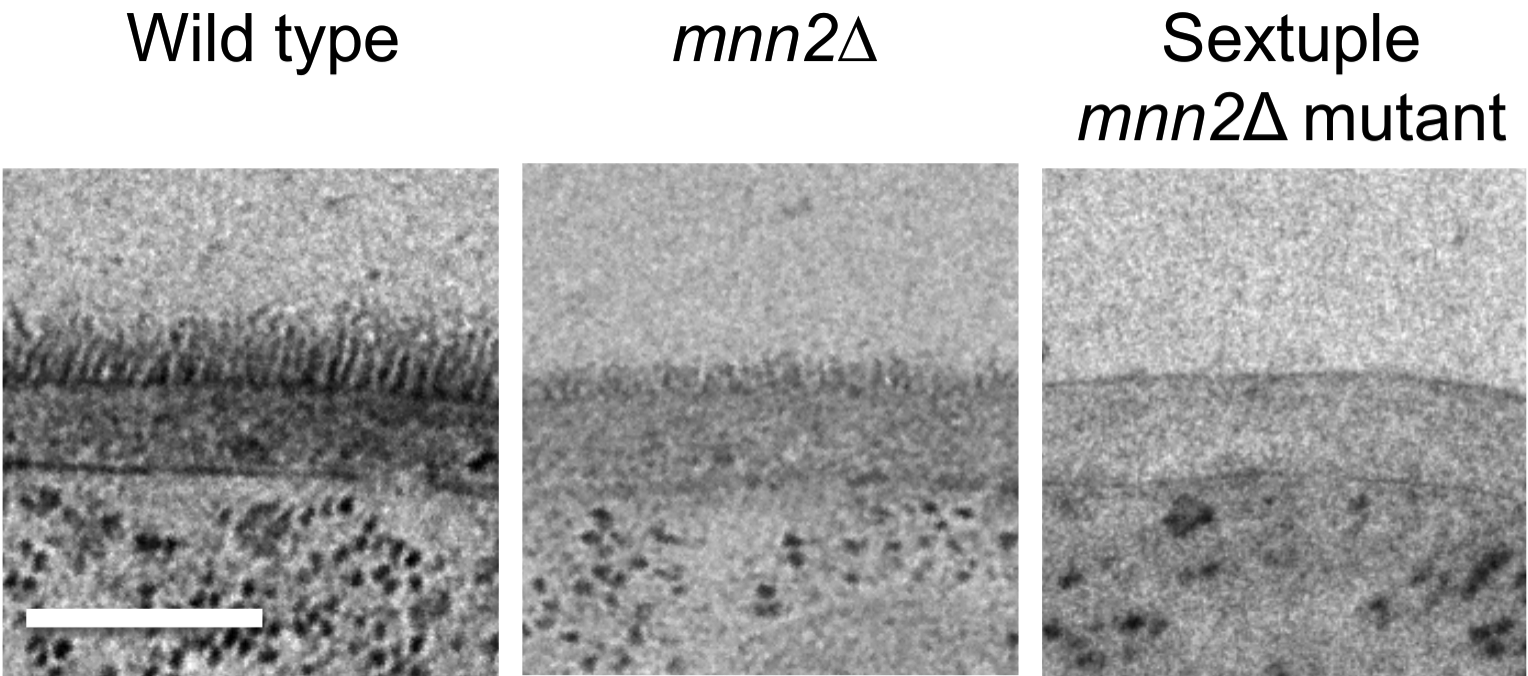
TEM of the cell wall in selected mannosylation mutants. The sextuple mnn2Δ mutant contains deletions in all six MNN2 genes (mnn2Δ/mnn21Δ/mnn22Δ/mnn23Δ/mnn24Δ/mnn26Δ). Uridine auxotrophy was complemented by the integration of the CIp10 plasmid at the RPS1 locus. The scale bar represents 0.2 μm.

The outer chain branched mannan is attached to the *N*‐mannan core through an α1,6‐backbone. Addition of the first α1,6‐mannose is catalysed by a single mannosyltransferase, Och1. Therefore, the *N*‐mannan of the *och1* mutant has no branched outer chain mannan, but the core *N*‐mannan contains additional mannose residues (Bates *et al*., 2006; Fig. 1). Deletion of *och1* results in significant shortening of the mannan fibrils (Netea *et al*., 2006), and the activation of the cell salvage pathway, resulting in an elevation in the levels of chitin and glucan, and hence a thickened cell wall (Bates *et al*., 2006). The α1,6‐mannose backbone is extended by the enzyme complexes mannan polymerase I (M‐Pol I) and mannan polymerase II (M‐Pol II). In *Saccharomyces cerevisiae,* M‐Pol I is composed of Mnn9 and Van1, while M‐Pol II is composed of Mnn9 and Anp1 (Hashimoto and Yoda, 1997; Jungmann and Munro, 1998). Deletion of the *C. albicans* Mnn9 orthologue results in a 50% decrease in total mannan levels, and a phenotype characterized by increased flocculation of yeast cells, reduced growth rates, osmotic sensitivity and abnormal morphogenesis (Southard *et al*., 1999). Therefore, it is likely that Mnn9 is the major contributor to the extension of the α1,6‐backbone in *C. albicans*. The backbone is then elaborated with extensive branches composed of α1,2‐mannose. In *S. cerevisiae,* the initial α1,2‐mannose unit is attached to the backbone via the actions of Mnn2, which are then extended with additional α1,2‐mannose units by Mnn5. blast searches of the *C. albicans* genome identify a family of related genes, which are putative Mnn2 and Mnn5 orthologues (Hall *et al*., 2013). Bai *et al*. characterized one of the family members and confirmed that the encoded protein had both α1,2‐ and α1,6‐mannosyltransferase activity, but was unable to complement the *S. cerevisiae mnn2*Δ mutant, and was hence designated an Mnn5 orthologue (Bai *et al*., 2006). A more detailed systematic characterization of this gene family suggests that three members have redundant Mnn2 activity, while the other three members display Mnn5‐like activity (Hall *et al*., 2013). The *C. albicans mnn5*Δ mutant also showed a reduced ability to synthesize *O*‐mannan (Bai *et al*., 2006). Deletion of Mnn2 and Mnn5 orthologues in *C. albicans* resulted in shortened mannan fibrils protruding from the cell wall, while deletion of all six genes abolished visible mannan fibrils (Fig. 2), with only α1,6‐mannose present in the *N*‐mannan side‐chain (Hall *et al*., 2013; Fig. 1). Biochemical evidence suggests that Mnt5 is also required for the addition of the second α1,2‐mannose unit to the outer chains from the *N*‐linked mannan (Díaz‐Jiménez *et al*., 2012), suggesting that there may be a degree of functional redundancy in the mannan biosynthetic pathways in *C. albicans.*

The α1,2‐mannnose chains are capped with α1,3‐mannose via the actions of Mnn1 (Yip *et al*., 1994; Romero *et al*., 1999). The *C. albicans MNN1* gene family contains 6 members, but only deletion of *MNN14* attenuates virulence (Bates *et al*., 2013), suggesting a degree of functional redundancy between family members. In contrast to *S. cerevisiae*, the *C. albicans N‐*mannan contains β1,2‐mannose, which forms part of both the acid‐stable and acid‐labile mannan fractions (see below), which are attached through the actions of β1,2‐mannosyltransferases (BMTs). Bmt1 and Bmt3 are required for the addition of the first and second β1,2‐mannose units respectively (Mille *et al*., 2008). However, removal of β1,2‐mannose from the acid‐stable mannan fraction did not affect growth, morphology or compromise the cell wall integrity (Mille *et al*., 2008). Therefore, the functional significance of β1,2‐mannosylation remains to be clarified. However β‐mannan plays important roles in immune recognition (see later).

#### Phosphomannan

The β1,2‐mannose moiety, linked to the branched *N*‐glycan through a phosphodiester bond, is commonly known as phosphomannan (PM), or acid‐labile mannan. Loss of this mannan fraction is characterized by a reduced ability of *C. albicans* to bind the cationic dye Alcian Blue, due to the loss of negative charge in the cell wall, as a result in the reduction of phosphate content. In *S. cerevisiae*, the PM is attached to the outer *N*‐mannan chains via Mnn4 and Mnn6 (Karson and Ballou, 1978; Nakayama *et al*., 1998). *ScMNN6* encodes the mannosylphosphate transferase (Odani *et al*., 1997), while ScMnn4 is a positive regulator of ScMnn6 (Odani *et al*., 1996). Deletion of the putative *C. albicans MNN4* orthologue impairs Alcian Blue binding to the *C. albicans* cell wall, confirming that it also participates in the attachment of PM to the outer *N*‐mannan chains (Hobson *et al*., 2004), although it has not been confirmed if CaMnn4 is acting as the mannosylphosphate transferase, or a positive regulator of CaMnn6. However, the *C. albicans mnn4*Δ mutant does maintain β1,2‐mannose in the acid‐stable fraction (Hobson *et al*., 2004; Singleton *et al*., 2005). The PM glycoconjugate is extended by a family of BMTs, which attach a series of β1,2‐mannose residues to the initial α1,2‐mannose. Bmt2, Bmt3 and Bmt4 are required for the addition of the first, second and third β1,2‐mannose units of the acid‐labile mannan respectively (Mille *et al*., 2008). Deletion of the α1,2‐mannosyltransferases *mnt3*Δ, and *mnt5*Δ together also results in reduced Alcian Blue binding (Mora‐Montes *et al*., 2010), suggesting they are also involved in elaboration/attachment of the PM to the *N*‐mannan, although *O*‐mannan can also incorporate PM. Removal of the PM, by deletion of *MNN4,* increases the net hydrophobicity of the cell wall (Singleton *et al*., 2005), and increases the resistance of the *N*‐mannan to acetolysis (Hazen *et al*., 2007), which cleaves α1,6‐linkages. This increased resistance suggests that Mnn4, in addition to regulating the addition of PM to the α1,2‐mannan side‐chain, may also have a global affect on the synthesis of acid‐stable mannan. The PM is important for macrophage phagocytosis (McKenzie *et al*., 2010). In comparison, removal of *O*‐ or *N*‐mannan resulted in increased phagocytosis (McKenzie *et al*., 2010), and increased exposure of β‐glucan, which would increase recognition through the phagocytic receptor Dectin‐1 (see below).

#### Other enzymes

The majority of the mannosyltransferases are metalloenzymes which require a metal ion cofactor [predominately manganese (Mn^2+^)] for functionality (Bai *et al*., 2006). Therefore, ion transport within the ER and Golgi network is an important factor for mannan biosynthesis. Pmr1 is a P‐type ATPase required for transporting divalent cations (Ca^2+^/Mn^2+^) into the Golgi and maintaining manganese homeostasis. Disruption of *PMR1* results in shortening of the branched *N*‐mannan and *O*‐mannan (Fig. 1), presumably due to the inhibition of several mannosyltransferases as a result of insufficient concentrations of cations within the Golgi (Bates *et al*., 2005). However, in comparison with the *och1*Δ mutant, the *pmr1*Δ has a thinner glucan‐chitin layer and longer, but less dense mannan fibrils.

### Phospholipomannan

Phospholipomannan (PLM) is comprised of mannosylated sphingolipids, sharing a mannose moiety similar to that of PM, composed of β1,2‐mannose, covalently linked to the lipid domain by a phosphodiester bond with an *α*‐mannose unit. Deletion of *MIT1* (Mannose Inositolphosphoceramide mannose Transferase) totally eliminated mannan from *C. albicans* PLM (Mille *et al*., 2004), suggesting that Mit1 is the sole transferase responsible for the addition of mannan to this lipid. The PLM is then elaborated with β‐mannose units, via the actions of Bmt5 and Bmt6 (Mille *et al*., 2012). Disruption of PLM significantly affected the *C. albicans* cell wall stress response due to calcium and SDS, but not Calcofluor White (Mille *et al*., 2004). Interestingly, blastospores shed PLM during early stages of macrophage phagocytosis, and the released PLM binds the surface of the macrophage (Jouault *et al*., 1998), where it participates in immune recognition of the fungal pathogen (see below).

In general, glycosylation mutants display similar phenotypes. For example, all glycosylation mutants studied, so far, show increased flocculation. For some of the mutants (*och1*Δ, *mnt1*Δ/*mnt2*Δ) this can be explained by a cell separation defect, at cytokinesis (Munro *et al*., 2005). However, this defect has not been observed for all the glycosylation mutants. One possible explanation is that the alterations to the glycosylation status of the cell wall affects the charge of the cell and hence the tendency to aggregate. It is also possible that the disruption of key regulatory cell wall processes affects the activity of glucanase and chitinase enzymes required for cell‐separation after cytokinesis. However, Gregori *et al*. recently showed that sub‐MIC concentrations of the β‐glucan synthase inhibitor caspofungin induce flocculation in an Efg1‐, Als1‐dependent manner, which could be inhibited by high concentrations of exogenous sugars (Gregori *et al*., 2011). Alternatively, overexpression of Als3 has been shown to induce flocculation. Expression of ALS proteins in the glycosylation mutants has not been studied, but evidence suggests that in addition to the glycome, the cell wall proteome is also altered in some mannosylation mutants, perhaps by inducing the unfolded protein response (Bates *et al*., 2005). Therefore, it is possible that manipulation of mannosylation alters many properties of the cell wall, which results in increased cell–cell adhesion, and could serve as an alternative mechanism for protection from the environment.

## Effects of the environment on mannan composition

The fungal cell wall is dynamic, and its composition is mediated by components of the surrounding environment. For example, the presence of echinocandin antifungals results in increased chitin synthesis to compensate for the depletion of glucan, to maintain cell wall integrity (Walker *et al*., 2008). Recent investigations into the mannan composition have shown that the environment also modulates the structure of the protruding mannan fibrils. At the molecular level, NMR data suggest that the structural composition of the mannan is dependent on growth conditions (Kruppa *et al*., 2011; Lowman *et al*., 2011). Growth in alternative carbon sources reduced chitin and glucan levels and also diminished the mannan fibrillar layer (Ene *et al*., 2012). Moreover, damage to the mannosylation structures upregulates *PMT1*, *PMT2* and *PMT4* in an Msb2‐, Cek1‐, Ace2‐dependent manner (Cantero and Ernst, 2011). Therefore, different growth conditions are likely to activate cell wall signalling cascades to varying degrees, altering the expression of cell wall biosynthesis genes, and affecting the mannan composition. For a detailed review of cell wall signalling pathways we direct readers to the following recent review (Ernst and Pla, 2011).

## Contribution of mannan to fungal immune recognition

Like many pathogens, *C. albicans* is detected and cleared predominantly through the actions of the innate immune system. Recognition of invading microbes is achieved by a variety of receptors on the surfaces of epithelia and myeloid cells. These include toll‐like receptors (TLRs), C‐type lectins (CTLs) and Nod‐like receptors (NDLs), which bind to specific epitopes on the pathogen surface (Medzhitov *et al*., 1997; Yang *et al*., 1998; Ariizumi *et al*., 2000). These so‐called pathogen recognition receptors (PRRs) and pathogen‐associated molecular patterns (PAMPs) now form the basis of our understanding of innate immune recognition. For example, TLR2, TLR4, dectin‐2, Mincle, DC‐SIGN and galectin‐3 have major roles in the recognition of fungal mannans (Fradin *et al*., 2000; Tada *et al*., 2002; Porcaro *et al*., 2003; Taylor *et al*., 2004; Rouabhia *et al*., 2005; McGreal *et al*., 2006), TLR9 recognizes fungal DNA (Miyazato *et al*., 2009), and dectin‐1 and complement receptor 3 (CR3) are the major PRRs involved in the detection of β‐glucans (Thornton *et al*., 1996; Brown and Gordon, 2001).

### Participation of *O*‐mannan to immune recognition

*O*‐mannan is predominately recognized by TLR4 (Netea *et al*., 2006). Deletion of TLR4 results in reduced neutrophil infiltration and enhanced fungal burden in the peritoneal exudates, lymph nodes and spleen (Gasparoto *et al*., 2010). Co‐incubation of oral epithelial cells with purified *C. albicans* cell wall components confirmed that these PAMPs only induced expression of TLR4, but epithelial cytokine production was independent of TLR4 (Wagener *et al*., 2012). However, a recent study highlighted that TLR4 recognition, is largely dependent on the *C. albicans* strain under investigation (Netea *et al*., 2010). In this study, the susceptibility of TLR4^−/−^ mice to *C. albicans* infection correlated with the dependence on TLR4 recognition, with disease progression unaltered in TLR4^−/−^ mice when infected with a *C. albicans* strain known to be independent of TLR4 recognition (Netea *et al*., 2010). Therefore, data regarding TLR4 recognition should be interpreted with caution.

### Participation of *N*‐mannan to immune recognition

*N*‐mannan is recognized by a multitude of receptors, which are expressed on different immune cells. The mannose receptor (MR) is an endocytic receptor thought to recognize terminal α1,2‐/α1,3‐mannose residues (Kéry *et al*., 1992; Netea *et al*., 2008). The MR is cleaved by a metalloproteinase producing a functional soluble (sMR) receptor (Martínez‐Pomares *et al*., 1998). The role of sMR in innate immunity has not been clarified, but the sMR may function to bind soluble mannan or degraded particles from phagocytosis events and present them to CR‐Fc^+^ cells surrounding the infection site (Martínez‐Pomares *et al*., 1998).

Due to the phagocytic nature of the MR, fungal cells with a low mannan content in their cell wall have reduced phagocytosis rates (Keppler‐Ross *et al*., 2010). Indeed, mutants with truncations in *N*‐ (*mns1*), phosphomannan (*mnn4*) and *O*‐linked mannan (*mnt1/mnt2*) exhibited delays in engulfment, but not in the rate of macrophage migration and chemotaxis towards *Candida* cells (Lewis *et al*., 2012). This is also true of neutrophils, where mannosylation mutants (for example, *och1*Δ, *pmr1*Δ and *mnt1*Δ/*mnt2*Δ) displayed a reduced phagocytosis index (Sheth *et al*., 2011). In neutrophils, at least, the decreased phagocytosis rate was found not to be due to lack of recognition, since neutrophils still had yeast bound to their surface. Instead, the reduced phagocytic index of the mannan‐deficient mutants seemed to be due to the failure of the neutrophils to engulf the mutants (Sheth *et al*., 2011). In contrast, alterations in other cell wall components, including glucan and chitin, did not markedly affect the efficiency of macrophages to phagocytose fungal cells (Keppler‐Ross *et al*., 2010). The MR is also responsible for the majority (70%) of dendritic cell (DC) recognition and internalization of *C. albicans* (Cambi *et al*., 2008). This recognition is mainly based on interactions with α1,2‐ or α1,3‐mannose, with the *och1*Δ and *pmr1*Δ mutants displaying reduce phagocytosis rates, while the *mnt1*Δ/*mnt2*Δ, *mnn4*Δ mutants, and the serotype B strains were still efficiently phagocytosed by DCs (Cambi *et al*., 2008).

Although the majority of *C. albicans* recognition by DCs occurs via the MR, DCs also express the C‐type lectin‐like receptor, DC‐SIGN. DC‐SIGN recognizes a variety of carbohydrate structures, including fructose and branched α‐mannan (Cambi *et al*., 2008), and can phagocytose *Candida* cells through the recognition of mannan (Cambi *et al*., 2003). The mouse orthologue of DC‐SIGN, SIGNR‐1, works in concert with Dectin‐1 to enhance the oxidative burst in macrophage cell lines (Takahara *et al*., 2011). Although DC‐SIGN and SIGNR‐1 are orthologues, they show distinct epitope specificity. For example, DC‐SIGN only recognizes α‐mannose residues with a free non‐reducing end (i.e. α‐mannose units at the end of the polymers), while SIGNR‐1 can also recognize α‐mannose units capped with additional α‐mannoses, or β‐mannose residues (Takahara *et al*., 2012).

In addition to the MR and DC‐SIGN, the C‐type lectin‐like receptor, dectin‐2 (Clec4n), has recently been identified as recognizing high mannose containing epitopes (> 7 terminal or branched α‐mannose residues) (McGreal *et al*., 2006), although the exact epitope (i.e. terminal, or branched α‐mannose units) recognized by dectin‐2 is unknown (Saijo *et al*., 2010). Deletion of dectin‐2 results in increased kidney fungal burdens and accelerated neutrophil infiltration, with *Candida* growth observed in the pelvis (Saijo *et al*., 2010), confirming that α‐mannan recognition via dectin‐2 is crucial for fungal detection and removal. Dectin‐2 recognition enhances secretion of IL‐1β, IL‐23 and IL‐6 and hence activates a protective Th17 response to the invading pathogen, as well as a less potent Th1 response (Saijo *et al*., 2010). In conjunction with this, *C. albicans* purified mannan is capable of inducing prostaglandin production from human PBMCs. β‐Glucan only enhanced prostaglandin levels in concert with TLR2 ligands (Smeekens *et al*., 2010). Furthermore, prostaglandin production is regulated via dectin‐2 and hence by mannan‐stimulation (Suram *et al*., 2010). Therefore, fungal mannan appears to play a critical role in inducing Th17 responses, presumably through the actions of CD14^++^/CD16^−^ subsets of circulating monocytes which have elevated expression of the MR on their surface (Smeekens *et al*., 2011), to fungal pathogens.

The β‐mannan which caps the branches of *N*‐mannan is recognized by galectin‐3 (Fradin *et al*., 2000). Although galectin‐3 can bind to a variety of β1,2‐epitopes, only recognition of antigenic factor 5 (phosphate bound β1,2‐mannose units) or factor 6 (terminal α1,3‐mannose units) exert fungicidal effects on *C. albicans.* These affects are specific for *Candida* species that display β1,2‐linked mannose on their surface, as galectin‐3 does not bind fungal cells that lack this epitope (for example *S. cerevisiae*) (Kohatsu *et al*., 2006). Macrophages isolated from galectin‐3 deficient mice exhibited normal levels of uptake and phagocytosis of *Candida* (Jouault *et al*., 2006), suggesting that recognition of β1,2‐mannan is not important for fungal eradication. However, more recently Linden *et al*. have shown that *Candida parapsilosis* induces galectin‐3 secretion from neutrophils, and propose that soluble galectin‐3 functions as a pro‐inflammatory autocrine/paracrine signal to enhance neutrophil phagocytosis (Linden *et al*., 2013).

In addition to the receptors described above, the C‐type lectin‐like receptor, Mincle which is expressed on macrophages, has been proposed to recognize α‐mannose units, but not complete mannan polysaccharides (Yamasaki *et al*., 2009). However, some conflicts exist in the literature regarding the role of Mincle in fungal infections. Mincle^−/−^ mice do not show increased susceptibility to systemic candidiasis, but they do display increased kidney burdens compared to control mice (Wells *et al*., 2008), suggesting that Mincle may play a role in fungal clearance. In agreement with this, TNFα secretion was reduced by 30% in Mincle^−/−^ bone marrow‐derived macrophages after stimulation with *C. albicans* (Wells *et al*., 2008). In contrast, Mincle specifically recognizes *Malassezia*, and not *C. albicans* or *Aspergillus* species (Yamasaki *et al*., 2009). The differences observed in this study might, in part, be attributed to the different *C. albicans* strains used in each study, which potentially has been attributed to the ability of different organisms to express different α‐mannose epitopes.

### Participation of phospholipomannan to immune recognition

Addition of purified PLM to macrophage‐like cells (J774) stimulates pro‐inflammatory cytokine secretion, suggesting that PLM contributes to innate immune recognition of *C. albicans* (Jouault *et al*., 1994; 1998). TLR knockout mice confirmed that PLM was recognized by TLR2, although bone marrow‐derived macrophages from TLR4^−/−^ and TLR6^−/−^ mice also showed reduced cytokine signalling in response to purified PLM, suggesting that these receptors may also function in the recognition of PLM (Jouault *et al*., 2003). However in keratinocytes, PLM induced pro‐inflammatory cytokine secretion (IL‐6 and IL‐8) was shown to be TLR2 dependent (Li *et al*., 2009). Therefore, the role of PLM in innate immune recognition may depend on the site of infection.

## Mannan and fungal diagnostics

Early detection of invasive candidaemia (IC) is essential for a good prognosis, with mortality rates increasing from 15% (antifungal treatment initiated immediately after positive blood culture), to 40% when treatment is delayed by 72 h (Garey *et al*., 2006). Despite the new developments in disease diagnostics, *Candida* infections are still hard to diagnose, with many cases going unreported until autopsy. Diagnosis is now based on the non‐invasive detection of circulating polysaccharides from the fungal cell wall in blood samples. Two of the diagnostic tests focus on circulating mannan levels, while the other is directed against β‐glucan.

### Mannan antigen detection

Mannan comprises up to 7% of the dry weight of *C. albicans* and is non‐covalently attached to the surface of the pathogen, and as a result is released into the circulation (Fukazawa, 1989). Therefore, patients with invasive candidaemia tend to have high circulating levels of mannan in their blood (mannanaemia). The first commercially available kit for the detection of mannan was Pastorex antigen agglutination kit, which gave varied results with a high percentage of false positives (Bailey *et al*., 1985; Lemieux *et al*., 1990). Currently, the conventional kit for testing sera for the presence of fungal mannan is the Platelia *Candida* antigen kit from Bio‐Rad, which is based on an enzyme‐linked immunosorbent assay (ELISA). The kit utilizes the rat monoclonal antibody EB‐CA1, which recognizes chains of α1,2‐mannose from the fungal cell wall in a size‐dependent manner, with five units being the minimum for efficient binding (Jacquinot *et al*., 1998). This assay assumes that mannan serum concentrations above 0.5 ng ml^−1^ are positive for candidaemia, and can lead to the identification of patients with candidaemia 7 weeks earlier than blood cultures (Nihtinen *et al*., 2011). The Platelia assay has a specificity of over 80% with a sensitivity of around 60% (Sendid *et al*., 1999; Alam *et al*., 2007; Mikulska *et al*., 2010; Mokaddas *et al*., 2011). However, increased sensitivity can be observed (70–100%) by decreasing the recommended cut‐off, but this increases false positives (Ellis *et al*., 2009; Mikulska *et al*., 2010). An alternative method is to use the assay in combination with another test like the anti‐mannan antibody detection kit (Arendrup *et al*., 2010; Mikulska *et al*., 2010). Initially there were concerns over the use of mannan as a diagnostic tool due to natural colonization of *Candida*. However, under these circumstances the mannan level remains within the cut‐off (i.e. below 0.5 ng ml^−1^), while they are greatly elevated in patients with invasive candidaemia (Mokaddas *et al*., 2010). Therefore, detection of mannan is a reliable diagnostic marker for invasive candidaemia. One factor that influences the accuracy of such diagnostics is the clearance of mannan from the circulation. Therefore, for high‐risk patients, such as those on immune suppressive therapy, or with neutropenia consistent monitoring of circulatory mannan levels may prove more beneficial than one‐off measurements.

### Anti‐mannan antibody detection

As discussed in the previous section, mannan is immune‐stimulatory and as a consequence antibodies are generated against it, the presence of which can then be used as a diagnostic tool to identify patients with fungal infections. The detection of anti‐mannan antibodies is taken advantage of in the Platelia *Candida* Ab assay kit. This assay involves the use of *Candida* mannan coated plates, to which sera from the patient is applied. The presence of the antibodies is achieved through a sandwich ELISA. Several studies have reported that the average sensitivity of the kit to detect patients infected with *Candida* is 60% with a range between 44% and 100%. However, the anti‐mannan test is less specific than the Platelia *Candida* antigen kit, due to high circulation of mannan antibodies from uninfected, but heavily colonized individuals (Odds and Evans, 1980), and the reduced antibody response in immune suppressed patients (Jones, 1990). It was reported that use of the anti‐mannan antibody test in combination with the mannan antigen test increases the sensitivity to 80–90% (Mikulska *et al*., 2010). Greater accuracy can also be achieved through the combined testing for *Candida* mannan and β‐glucan, or *Candida* mannan, β‐glucan and *Candida* DNA (Alam *et al*., 2007). The use of these biological markers to detect IC in high‐risk patients has proven successful in the early detection of infection, producing positive results up to 7 days before a positive blood culture.

### Other fungal species

Although much of the knowledge we have on the fungal cell wall has been based on studies from *S. cerevisiae* and *C. albicans,* which have similar cell wall structures, new insights are now coming from studies of other pathogenic fungi. These studies confirm that the structural organization of some elements of the fungal cell wall are well conserved, with most fungi having a common core comprised of chitin and β‐glucan in the inner wall layer and an outer layer of glycoproteins. The ratio of the components and the major carbohydrate components and the amount of glycoprotein in the wall vary significantly. For example, chitin forms only 2–5% of the dry weight of the *C. albicans* cell wall, while it accounts for over 10–20% of the dry weight of the walls of *Aspergillus* or *Neurospora* species. In *Aspergillus* species, the glucan layer is comprised of β1,3‐ and β1,4‐glucan, while *C. albicans* contains β1,3‐ and β1,6‐glucan (Fontaine *et al*., 2000). Some fungi have considerably less glycoproteins in their cell wall than *C. albicans,* and these proteins are glycosylated with polysaccharide structures other than mannan. In *Aspergillus fumigatus*, and *Malassezia furfur* the glycoproteins are glycosylated with polysaccharides composed of mannose and galactose monosaccharides, known as galactomannan (Latgé *et al*., 1994; Shibata *et al*., 2009), and circulating galactomannan levels are the most commonly used diagnostic marker for invasive aspergillosis (Rohrlich *et al*., 1996). In addition, long complex glycosylation structures such as the *N*‐mannan in *C. albicans* are not present in filamentous fungi, but instead *N*‐mannans are often shorter and terminate in galactofuranose (Leitão *et al*., 2003; Morelle *et al*., 2005). In some fungi, a polysaccharide capsule surrounds the cell wall. *Cryptococcus neoformans* and *C. gattii* are surrounded by a glucuronoxylomannan (GXM) and galactoxylomannan (GalXM) capsule, which forms a physical barrier protecting the fungus from the environment and host immune defences (O'Meara and Alspaugh, 2012). The capsule is also a major diagnostic marker, which can be visualized by India ink staining, or quantified through the detection of Cryptococcal antigen (CrAg) by latex aggregation, ELISA or lateral flow (Kozel and Bauman, 2012; O'Meara and Alspaugh, 2012).

## Conclusions

The fungal cell wall is a dynamic structure important for maintaining cell shape, protection against environmental stress and immune recognition. The outer most layer of the fungal cell wall is comprised of glycosylated proteins, the carbohydrate structures of which serve as PAMPs that trigger immune recognition. A series of glycosylation mutants, which express altered mannan epitopes on the cell surface, have shed light on the role of different mannans in fungal immune recognition. Many of these mutants show similar phenotypic characteristics including increased flocculation, decreased growth rates, abnormal morphogenesis, temperature sensitivity, increased sensitivity to cell wall perturbing agents and a reduced ability to active host immune responses, all of which result in attenuated virulence. However, immune responses are dependent on the type of immune cell. For example, the mutants which are defective in mannan (*och1*Δ, *mnt1*Δ/*mnt2*Δ and *mns1*Δ) show a reduced ability to activate peripheral blood monocytes (Munro *et al*., 2005; Bates *et al*., 2006; Mora‐Montes *et al*., 2007), but are phagocytosed by macrophages at a higher rate than wild type (McKenzie *et al*., 2010), suggesting that recognition in monocytes is predominately driven by mannan through the TLR4 and the MR, while macrophage recognition is predominately mediated by β‐glucan, through dectin‐1. Moreover, during tissue invasion, where fungal β‐glucan exposure is increased, the immune stimulation becomes more dependent on β‐glucans (Wheeler *et al*., 2008). It is also important to consider that local host environmental signals can strongly influence cell wall structure and composition and so immune recognition of the wall is presented with a moving target (Kruppa *et al*., 2011; Lowman *et al*., 2011). During the infection process, *C. albicans* will be exposed to a plethora of signals including environments of different pH and CO_2_ levels, different carbon sources (Ene *et al*., 2012), etc., all of which may individually or simultaneously impact on the cell wall altering the way in which the immune system sees the fungus. The affect of host environmental cues on the fungal cell wall is currently an understudied area of fungal biology, but this area is important if we want to fully understand the extent of the interactions that occur between the host and pathogen during infection.
